# Decrease in waist-to-hip ratio reduced the development of chronic kidney disease in non-obese non-alcoholic fatty liver disease

**DOI:** 10.1038/s41598-020-65940-y

**Published:** 2020-06-02

**Authors:** Young Eun Chon, Hyung Jong Kim, Yu Bum Choi, Seong Gyu Hwang, Kyu Sung Rim, Mi Na Kim, Joo Ho Lee, Yeonjung Ha, Mi Jung Lee

**Affiliations:** 1 Division of Gastroenterology, Department of Internal Medicine, Institute of Gastroenterology, CHA Bundang Medical Center, CHA University, Seongnam, Korea; 2 Department of Internal Medicine, CHA Bundang Medical Center, CHA University, Seongnam, Korea

**Keywords:** Gastroenterology, Nephrology

## Abstract

To date, there are few studies that have evaluated the prognostic impact of changes in abdominal obesity or weight on long-term adverse kidney outcomes in non-alcoholic fatty liver disease (NAFLD). We investigated the effect of changes in waist-to-hip ratio (WHR) and body weight (BW) on chronic kidney disease (CKD) development, especially in non-obese NAFLD patients. We included 6,137 participants from a community-based prospective cohort with 12-year follow-up in Korea. NAFLD patients were categorized according to time-averaged percent changes in WHR and BW (≤−5%, >−5% to <5%, and ≥5%). Compared to non-obese controls, non-obese NAFLD was significantly associated with an increased risk of incident CKD (hazard ratio [HR] = 1.238, 95% confidence interval [CI] = 1.006–1.524). In 1,563 NAFLD patients, compared to patients with minimal changes in WHR (>−5% to <5%), patients with a decreased WHR (≤−5%) had a significantly attenuated risk of CKD development (HR = 0.300; 95% CI = 0.194–0.464). Furthermore, risk reduction from decreased WHR for developing CKD remained significant in non-obese NAFLD patients (HR = 0.290; 95% CI = 0.114–0.736). In conclusion, a decrease in WHR of more than 5% significantly reduced the risk of CKD development in NAFLD patients, even in those who were non-obese. Thus, serial monitoring of WHR may be prioritized in the management of NAFLD.

## Introduction

Non-alcoholic fatty liver disease (NAFLD) is a major chronic liver disease and a common cause of hepatocellular carcinoma and liver transplantation^1,2^. The incidence and prevalence of NAFLD have continuously increased, and are a growing concern for global public health^3^. Obesity is an established key factor for development and progression of NAFLD^4^. However, NAFLD can develop even in a patient who is not obese, known as “non-obese NAFLD”^5–10^. Several studies have examined the metabolic profile or cardiovascular outcomes of non-obese or lean NAFLD patients, but have shown mixed results^5–16^. Meanwhile, emerging evidence has revealed that NAFLD is implicated in not only liver-related complications but also extra-hepatic complications including diabetes, cardiovascular disease, and chronic kidney disease (CKD)^3,17–21^. Although CKD is an important extra-hepatic complication of NAFLD^17,19–21^, the clinical implication of non-obese NAFLD on kidney function has not been fully explored. Therefore, in the present study, we explored the metabolic characteristics of patients with non-obese NAFLD compared to obese NAFLD and non-obese healthy participants and determined the prognostic impact of non-obese NAFLD on adverse kidney outcomes.

To date, significant and sustained weight reduction is a crucial treatment strategy in patients with NAFLD^22–24^ and is also considered important in non-obese NAFLD patients^9,10,15,25^. The beneficial effect of weight reduction has been explained by its close association with regional body fat distribution, especially visceral fat^9,10,13^. Moreover, a recent study demonstrates that patients with non-alcoholic steatohepatitis treated with a year of lifestyle modification including weight reduction  were associated with an improvement in liver histology and kidney function^20^. These findings led us to investigate whether changes in abdominal fat can affect the risk of long-term adverse kidney outcomes in non-obese NAFLD patients. To address this issue, we explored the independent association between changes in waist-to-hip ratio (WHR), a useful anthropometric index for central obesity, and CKD development in non-obese NAFLD patients using a large scale, community-based, prospective cohort with a 12-year follow-up in Korea.

## Results

### Baseline characteristics of study participants

Baseline characteristics of study participants are shown in Table 1. The mean age was 51.5 ± 8.6 years and 2,716 participants (44.3%) were men. Among the 6,137 participants, 25.5% had NAFLD, and the mean estimated glomerular filtration rate (eGFR) was 93.0 ± 13.1 mL/min/1.73 m^2^. The mean body mass index (BMI) was 24.5 ± 3.1 kg/m^2^ and 3,554 participants (57.9%) were non-obese. The overall prevalence of non-obese NAFLD was 7.5% (459/6,137). When obese and non-obese participants were considered respectively, the prevalence of NAFLD was 12.9% (459/3,554) in non-obese participants and 42.7% (1,104/2,583) in obese participants.

**Table 1:** Baseline characteristics of participants.

	All(n = 6,137)	Non-obese	Obese	P
		(BMI < 25 kg/m^2^)	(BMI ≥ 25 kg/m^2^)	
		(n = 3,554)	(n = 2,583)	
Age, years	51.5 ± 8.6	51.5 ± 8.8	51.5 ± 8.2	0.9
Men, n (%)	2,716 (44.3%)	1,638 (46.1%)	1,078 (41.7%)	<0.001
Education, n (%)				<0.001
≤ 6th grade	1,864 (30.4%)	1,014 (28.5%)	850 (32.9%)	
7th to 12th grade	3,411 (55.6%)	2,048 (57.6%)	1,363 (52.8%)	
>12th grade	862 (14.0%)	492 (13.8%)	370 (14.3%)	
Income, n (%)				0.10
<$1,000/m	1,919 (31.3%)	1,150 (32.4%)	769 (29.8%)	
$1,000 to $2,000/m	1,917 (31.2%)	1,094 (30.8%)	823 (31.9%)	
>$2,000/m	2,301 (37.5%)	1,310 (36.9%)	991 (38.4%)	
Current smoker, n (%)	2,260 (36.8%)	1,373 (38.6%)	887 (34.3%)	<0.001
Diabetes mellitus, n (%)	219 (3.6%)	99 (2.8%)	120 (4.6%)	<0.001
Hypertension, n (%)	794 (12.9%)	288 (8.1%)	506 (19.6%)	<0.001
†CVD, n (%)	139 (2.3%)	75 (2.1%)	64 (2.5%)	0.34
MS, n (%)	2.123 (34.6%)	686 (19.3%)	1,437 (55.6%)	<0.001
Body weight, kg	62.7 ± 9.9	57.8 ± 7.5	69.5 ± 8.7	<0.001
BMI, kg/m^2^	24.5 ± 3.1	22.5 ± 1.8	27.3 ± 2.0	<0.001
WC, cm	84.5 ± 8.5	79.6 ± 5.8	91.2 ± 6.9	<0.001
HC, cm	93.7 ± 5.8	90.8 ± 4.7	97.6 ± 4.8	<0.001
WHR	0.90 ± 0.05	0.87 ± 0.03	0.93 ± 0.04	<0.001
MAP, mmHg	92.8 ± 12.7	90.6 ± 12.3	95.8 ± 12.6	<0.001
Platelet, x10^3^/μL	266.9 ± 62.4	262.2 ± 61.1	273.3 ± 63.6	<0.001
Creatinine, μmol/L	73.3 ± 14.6	72.8 ± 14.3	74.0 ± 15.1	0.002
Albumin, g/L	43 ± 3	43 ± 3	43 ± 3	0.78
AST, IU/L	26.0 (22.0–31.0)	25.0 (22.0–30.0)	26.0 (23.0–31.0)	<0.001
ALT, IU/L	22.0 (17.0–30.0)	20.0 (16.0–27.0)	25.0 (19.0–34.0)	<0.001
Total bilirubin, μmol/L	10.3 ± 5.1	10.3 ± 5.1	10.3 ± 5.1	0.67
GGT, U/L	17.0 (12.0–30.0)	15.0 (11.0–26.0)	21.0 (13.0–36.0)	<0.001
Fasting glucose, mmol/L	4.8 ± 1.0	4.7 ± 1.0	4.9 ± 1.1	<0.001
Fasting insulin, pmol/L	6.9 (5.2–9.5)	6.3 (4.8–8.4)	8.0 (5.8–10.5)	<0.001
HOMA-IR	1.62 ± 1.13	1.43 ± 0.97	1.90 ± 1.27	<0.001
Total cholesterol, mmol/L	5.0 ± 0.9	4.9 ± 0.9	5.1 ± 0.9	<0.001
HDL-C, mmol/L	1.2 ± 0.3	1.2 ± 0.3	1.1 ± 0.2	<0.001
LDL-C, mmol/L	3.1 ± 0.9	3.0 ± 0.8	3.2 ± 0.9	<0.001
Triglyceride, mmol/L	1.8 ± 1.1	1.6 ± 1.0	2.0 ± 1.2	<0.001
CRP, nmol/L	0.12 (0.06–0.23)	0.12 (0.05–0.20)	0.16 (0.08–0.27)	<0.001
eGFR, mL/min/1.73m^2^	93.0 ± 13.1	93.8 ± 12.9	92.0 ± 13.2	<0.001
NFLD, n (%)	1,563 (25.5)	459 (12.9%)	1,104 (42.7%)	<0.001

Note: Data are expressed as the mean ± standard deviation, median (interquartile range), or number of patients (percent).

†CVD: A composite of CAD, PAD, CVA, and CHF

Abbreviations: ALT, alanine aminotransferase; AST, aspartate aminotransferase; BMI, body mass index; CAD, coronary artery disease; CHF, congestive heart failure; CRP, C-reactive protein; CVA, cerebrovascular accident; CVD, cardiovascular disease; eGFR, estimated glomerular filtration rate; GGT, gamma glutamyl transferase; HC, hip circumference; HDL-C, high-density lipoprotein cholesterol; HOMA-IR, homeostatic model assessment of insulin resistance; LDL-C, low-density lipoprotein cholesterol; MAP, mean arterial pressure; MS, metabolic syndrome; NAFLD, non-alcoholic fatty liver disease; PAD, peripheral artery disease; WC, waist circumference; WHR, waist-to-hip ratio.

### Metabolic profiles of non-obese NAFLD patients

We compared clinical and metabolic characteristics between the non-obese NAFLD and obese NAFLD groups (Table 2). The WHR, blood pressure, total cholesterol, low density lipoprotein cholesterol, and C-reactive protein concentrations were lower in the non-obese NAFLD than obese NAFLD group, but homeostatic model assessment of insulin resistance (HOMA-IR) was similar. Approximately 80% of non-obese NAFLD patients had metabolic syndrome (MS), which was a significantly higher prevalence than in the non-obese controls (10.1%, P < 0.001) (Supplementary Table 1). Furthermore, non-obese NAFLD patients had greater values across the all individual components of MS, including WHR, blood pressure, fasting glucose, lipid profiles, and HOMA-IR than in the non-obese controls. In terms of baseline kidney function, eGFR was not different between non-obese NAFLD and non-obese controls, but was higher than the obese NAFLD group.

**Table 2:** Comparison of metabolic characteristics between non-obese NAFLD and obese NAFLD patients.

	All NAFLD	Non-obese NAFLD	Obese NAFLD	P
	(n = 1,563)	(n = 459)	(n = 1,104)	
Age, years	52.8 ± 8.3	53.9 ± 8.5	52.3 ± 8.2	<0.001
Men, n (%)	710 (45.4%)	213 (46.4%)	497 (45.0%)	0.62
DM, n (%)	176 (11.3%)	67 (14.6%)	109 (9.9%)	0.01
IFG or DM, n (%)	408 (26.1%)	124 (27.0%)	284 (25.7%)	0.32
Hypertension, n (%)	397 (25.4%)	86 (18.7%)	311 (28.2%)	<0.001
Dyslipidemia, n (%)	1224 (78.3%)	360 (78.4%)	864 (78.3%)	0.50
MS, n (%)	1,381 (88.4%)	372 (81.0%)	1,009 (91.4%)	<0.001
BMI, kg/m^2^	26.5 ± 2.9	23.3 ± 1.4	27.9 ± 2.2	<0.001
WHR	0.93 ± 0.04	0.89 ± 0.03	0.94 ± 0.04	<0.001
MAP, mmHg	99.1 ± 11.6	97.4 ± 11.5	99.9 ± 11.6	<0.001
AST, IU/L	29.0 (24.0–36.0)	29.0 (25.0–37.0)	29.0 (24.0–35.0)	0.11
ALT, IU/L	30.0 (23.0–45.0)	29.0 (22.0–45.0)	31.0 (24.0–45.0)	0.26
Total bilirubin, μmol/L	10.3 ± 5.1	10.3 ± 5.1	10.3 ± 5.1	0.18
FIB-4	0.93 (0.73–1.23)	0.99 (0.79–1.30)	0.91 (0.71–1.20)	<0.001
GGT, U/L	27.0 (16.0–49.0)	25.0 (14.0–53.0)	27.5 (17.0–49.0)	0.15
Fasting glucose, mmol/L	5.2 ± 1.5	5.2 ± 1.8	5.2 ± 1.4	0.9
HOMA-IR	2.51 ± 1.71	2.42 ± 1.97	2.55 ± 1.58	0.17
Total cholesterol, mmol/L L	5.2 ± 0.9	5.0 ± 0.9	5.2 ± 0.9	<0.001
HDL-C, mmol/L	1.0 ± 0.2	1.1 ± 0.3	1.0 ± 0.2	0.05
LDL-C, mmol/L	3.2 ± 0.9	3.0 ± 0.9	3.2 ± 0.9	<0.001
Triglyceride, mmol/L	2.5 ± 1.5	2.5 ± 1.7	2.5 ± 1.4	0.69
CRP, mmol/L	0.18 (0.10–0.29)	0.16 (0.08–0.27)	0.19 (0.11–0.30)	<0.001
eGFR, mL/min/1.73m^2^	91.9 ± 12.9	93.2 ± 12.4	91.4 ± 13.1	0.01

Note: Data are expressed as the mean ± standard deviation, median (interquartile range), or number of patients (percent).

Abbreviations: ALT, alanine aminotransferase; AST, aspartate aminotransferase; BMI, body mass index; CRP, C-reactive protein; DM, diabetes mellitus; eGFR, estimated glomerular filtration rate; FIB-4, fibrosis-4; GGT, gamma glutamyl transferase; HDL-C, high-density lipoprotein cholesterol; HOMA-IR, homeostatic model assessment of insulin resistance; IFG, impaired fasting glucose; LDL-C, low-density lipoprotein cholesterol; MAP, mean arterial pressure; MS, metabolic syndrome; NAFLD, non-alcoholic fatty liver disease; WHR, waist-to-hip ratio.

### Risk of incident CKD in non-obese NAFLD

During a mean follow-up duration of 116.3 ± 40.3 months, CKD developed in 1,258 patients (20.5%) (Table 3). The incidence rate of CKD was the lowest (1.79 per 100 person-years; 17.7%) in the non-obese control group and the highest (3.06 per 100 person-years; 28.5%) in the obese NAFLD group. In the non-obese NAFLD group, CKD developed in 26.1% patients and the incidence rate of CKD was 2.83 per 100 person-years. Compared to the non-obese controls, non-obese NAFLD patients had a significantly higher risk of CKD development (hazard ratio [HR] = 1.238; 95% confidence interval [CI] = 1.006–1.524; P = 0.04) as well as obese NAFLD patients (HR = 1.330; 95% CI = 1.142–1.549; P < 0.001).

**Table 3:** Uni- and multivariable Cox regression analyses for risk of CKD development according to obesity and NAFLD.

	No. of events (%)	Event rate per 100 P-Y	Crude	P	Fully adjusted	P
			HR (95% CI)		HR (95% CI)	
Non-obese control	547 (17.7%)	1.79	1 (reference)		1 (reference)	
Obese control	276 (18.7%)	1.92	1.069 (0.925–1.236)	0.37	1.005 (0.867–1.163)	0.9
Non-obese NAFLD	120 (26.1%)	2.83	1.608 (1.320–1.959)	<0.001	1.238 (1.006–1.524)	0.04
Obese NAFLD	315 (28.5%)	3.06	1.723 (1.500–1.980)	<0.001	1.330 (1.142–1.549)	<0.001

Note: Fully adjusted models included age, sex, education levels, income levels, smoking status, diabetes mellitus, hypertension, dyslipidemia, history of CVD, CRP concentrations, and baseline eGFR

Abbreviations: CI, confidence interval; CKD, chronic kidney disease; CRP, C-reactive protein; CVD, cardiovascular disease; eGFR, estimated glomerular filtration rate; HR, hazard ratio; NAFLD, non-alcoholic fatty liver disease; P-Y, person-year.

### Effect of changes in WHR on CKD development

**Figure 1:**
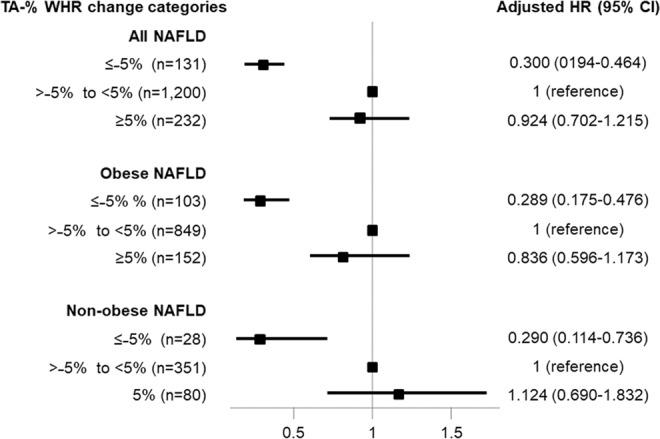
Adjusted hazard ratios of the TA-% WHR change categories for CKD development in NAFLD patients. Adjusted HR was determined by multivariable Cox regression analysis after age, sex, education, income, smoking status, diabetes mellitus, hypertension, dyslipidemia, history of cardiovascular disease, C-reactive protein concentrations, and baseline eGFR adjustment. Stratified analyses were performed in obese NAFLD and non-obese NAFLD patients, respectively. Abbreviation: CI, confidence interval; CKD, chronic kidney disease; eGFR, estimated glomerular filtration rate; HR, hazard ratio; NAFLD, non-alcoholic fatty liver disease; TA-% WHR change, time-averaged percent waist-to-hip ratio change.

NAFLD patients were categorized into three groups according to time averaged percent WHR change (TA-% WHR change) or time averaged percent body weight change (TA-% BW change) (≤−5%, >−5% to <5%, and ≥5%), respectively (Supplementary Table 2). To ascertain the independent association between WHR changes and CKD development in NAFLD, multivariable Cox regression analyses were performed (Fig. 1). In all NAFLD patients, compared to patients with minimal changes in WHR (>−5% to <5%), patients with TA-% WHR change ≤−5% showed a 70% lower risk of CKD development (HR = 0.300; 95% CI = 0.194–0.464; P < 0.001). Moreover, a significant risk reduction from decreased WHR (TA-%WHR change ≤ −5%) for developing CKD remained in non-obese NAFLD patients (HR = 0.290; 95% CI = 0.114–0.736; P = 0.01), as well as obese NAFLD patients (HR = 0.289; 95% CI = 0.175–0.476; P < 0.001).

### Effect of changes in BW on CKD development

**Figure 2:**
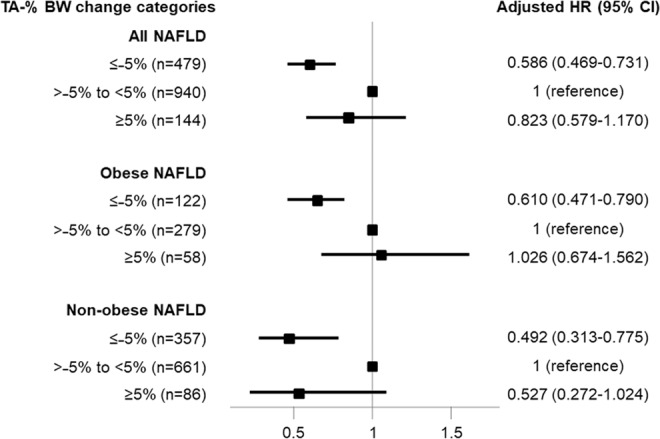
Adjusted hazard ratios of the TA-% BW change categories for CKD development in NAFLD patients. Adjusted HR was determined using multivariable Cox regression analysis after adjustment for age, sex, education, income, smoking status, diabetes mellitus, hypertension, dyslipidemia, history of cardiovascular disease, C-reactive protein concentration, and baseline eGFR. Stratified analyses were performed in obese NAFLD and non-obese NAFLD patients, respectively. Abbreviations: CI, confidence interval; CKD, chronic kidney disease; eGFR, estimated glomerular filtration rate; HR, hazard ratio; NAFLD, non-alcoholic fatty liver disease; TA-% BW change, time-averaged percent body weight change.

To test the independent effect of changes in BW on incident CKD in NAFLD patients, multivariable Cox regression analyses were performed (Fig. 2). NAFLD patients with a BW decrease more than 5% had a significantly decreased risk of CKD, compared with the minimal change group (HR = 0.586; 95% CI = 0.469–0.731; P < 0.001; >−5% to <5% as reference). Furthermore, decreased risk for incident CKD from a mean weight loss of more than 5% was significant in both the obese NAFLD group (HR = 0.610; 95% CI = 0.471–0.790; P < 0.001) and the non-obese NAFLD group (HR = 0.492, 95% CI = 0.313–0.775; P = 0.002).

### Subgroup analysis

**Figure 3:**
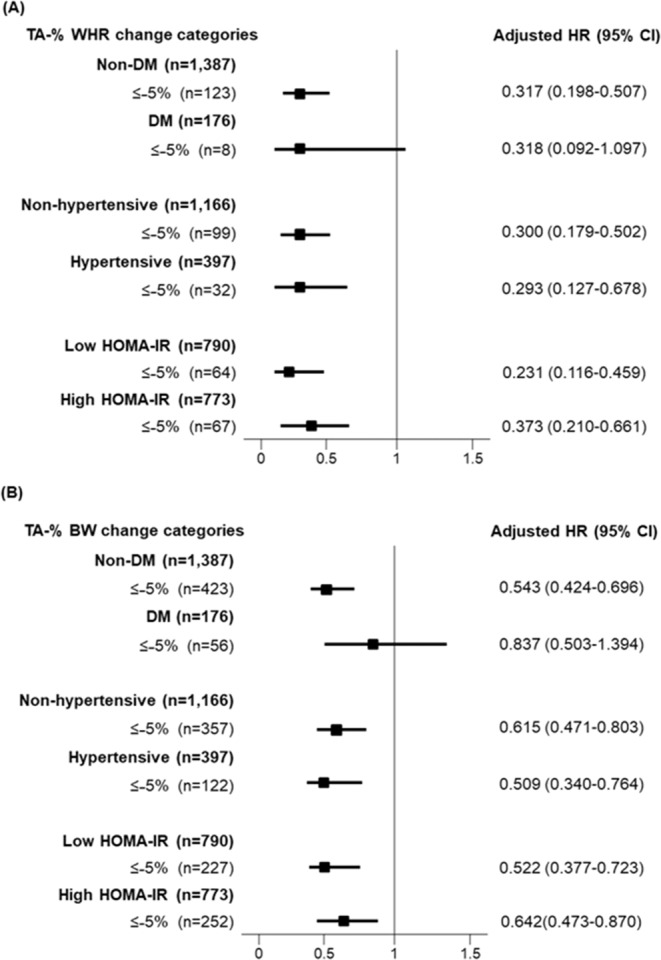
Adjusted HRs of the decreased TA-% WHR change and TA-% BW change for incident CKD in NAFLD patients according to subgroups of DM, hypertension, and HOMA-IR. Adjusted HRs of TA-% WHR (≤−5%) (**A**) and TA-% BW (≤−5%) (**B**) were calculated after adjustment for age, sex, education, income, smoking status, DM, hypertension, dyslipidemia, history of cardiovascular disease, C-reactive protein concentrations, and baseline eGFR. HOMA-IR group was dichotomized by the median value of HOMA-IR. Abbreviation: CI, confidence interval; CKD, chronic kidney disease; eGFR, estimated glomerular filtration rate; HOMA-IR, homeostatic model assessment of insulin resistance; hazard ratio; NAFLD, non-alcoholic fatty liver disease; TA-% WHR change, time-averaged percent waist-to-hip ratio change.

Subgroup analyses were performed according to diabetes mellitus, hypertension, and insulin resistance. NAFLD patients were dichotomized by the median value of HOMA-IR, 2.17. Risk reduction from decreased WHR (TA-% WHR change ≤ −5%) or BW (TA-% BW change ≤ −5%) was consistently found in the non-diabetic, non-hypertensive, hypertensive, low HOMA-IR, and high HOMA-IR groups (Fig. 3).

### Changes in insulin resistance according to WHR and BW changes

**Figure 4:**
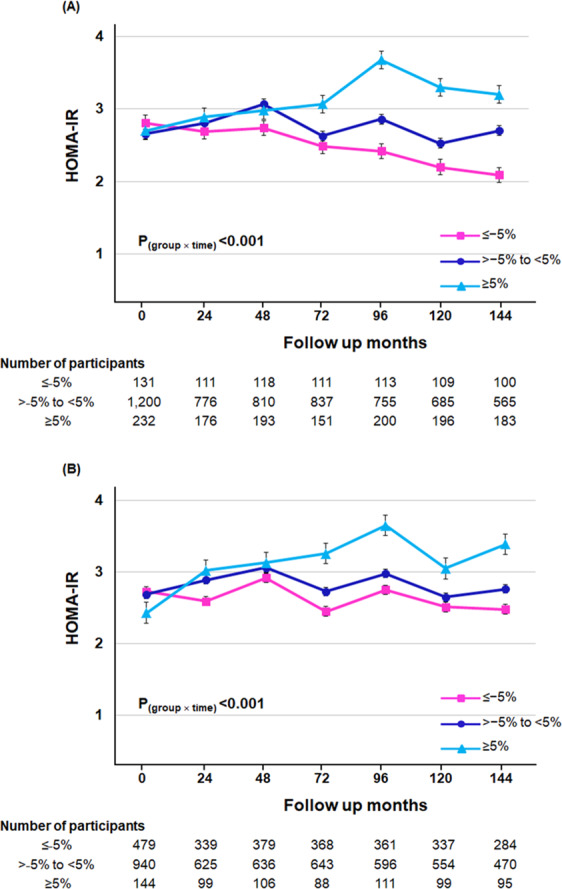
The trajectory of HOMA-IR over the follow-up period according to the TA-% WHR change and TA-% BW change in NAFLD patients. Mean HOMA-IR with standard error was depicted based on categorization of (**A**) TA-% WHR change and (**B**) TA-% BW change. P value was calculated by mixed-effects model. Abbreviations: HOMA-IR, homeostatic model assessment of insulin resistance; NAFLD, non-alcoholic fatty liver disease; TA-% BW change, time-averaged percent body weight change; TA-% WHR change, time-averaged percent WHR change.

To verify the effect of WHR or BW changes on insulin resistance, we calculated the slope of HOMA-IR changes during follow-up (ΔHOMA-IR per year) according to TA-% WHR or BW categories (Supplementary Table 3). HOMA-IR did not differ among the three TA-% WHR change groups at baseline (P = 0.63). HOMA-IR decreased in patients with a mean WHR decrease of more than 5% during follow-up (mean [95% CI]; −0.04 [−0.09 to 0.02] per year), but increased in the other two groups (0.04 [0.01 to 0.06] per year in TA-% WHR change > −5% to <5%; 0.19 [0.05 to 0.28] in TA-% WHR change ≥5%). Furthermore, there were significant differences in the changes in HOMA-IR during the follow-up period among the three groups (P _(group × time)_ <0.001) (Fig. 4). This trend was also observed in non-obese and obese NAFLD patients (both P _(group × time)_ <0.001) (Supplementary Table 3). In terms of BW changes, HOMA-IR did not decrease in patients with a more than 5% reduced BW (0.01 [−0.02 to 0.05] per year), but there was significant differences in HOMA-IR changes during the follow-up period among the three TA-% BW change groups (P _(group × time)_ <0.001) (Fig. 4).

## Discussion

In the present study, we demonstrated that not only obese NAFLD patients, but also non-obese NAFLD patients, had a significantly greater risk of CKD development. Furthermore, NAFLD patients with a decreased WHR of more than 5% during follow-up had a significantly attenuated risk of incident CKD. Of note, a significant risk reduction for CKD development provided by reduced WHR was evident in non-obese NAFLD as well as obese NAFLD patients. These findings suggest that sustained reduction of abdominal fat may be a useful strategy to prevent adverse kidney outcomes in NAFLD patients, even in those who are non-obese.

Obesity is closely implicated in the pathogenesis of NAFLD^4^. However, NAFLD can occur in subjects who are not obese (by region-specific BMI cutoff, BMI < 25 kg/m^2^ in Asian populations; <30 kg/m^2^ in non-Asian populations), which is known as “non-obese NAFLD”^6–10^. The prevalence of non-obese NAFLD in Korea ranged from 12.6% in a community-based study^5^ to 27% in a health examination cohort^6^. The prevalence of non-obese NAFLD in this study was 12.9% among non-obese participants, which is similar to a previous community-based study^5^. In the current study, compared to obese NAFLD, non-obese NAFLD patients had a lower values for metabolic risk factors, including WHR, blood pressure, lipid profile, and C-reactive protein concentration. However, the majority of non-obese NAFLD patients had MS (approximately 80%), and all individual components of MS were significantly higher than in the non-obese controls. These findings were in line with a recent meta-analysis^16^. Sookoian and Pirola^16^ indicated that lean NAFLD patients (BMI < 23 kg/m^2^ in Asian populations) shared a common altered metabolic and cardiovascular profile with obese NAFLD patients, and they exhibited a greater metabolic risk compared with lean controls.

Emerging evidence demonstrates that NAFLD is closely associated with the development of CKD^17,19–21^. A meta-analysis including nine observational studies of 96,595 adults revealed that patients with NAFLD had a higher risk of incident CKD than those without NAFLD^21^. Obese NAFLD patients can be easily predicted to have a greater CKD risk, because of their profound metabolic risk. However, the implication of non-obese NAFLD on adverse kidney outcomes has not been fully explored. In this study, we found that not only obese NAFLD, but also non-obese NAFLD patients, had a significantly higher risk of incident CKD compared with non-obese controls. There are several possible reasons that underlie the increased risk of CKD in non-obese NAFLD patients. First, our non-obese NAFLD patients had a high prevalence of MS. Because CKD is significantly associated with MS^26^, CKD may develop as a consequence of the adverse effects of MS, including insulin resistance^27^. In our study, HOMA-IR, a surrogate for insulin resistance, was greater in patients with non-obese NAFLD than non-obese controls (Supplementary Table 1, 2.42 ± 1.96 vs. 1.28 ± 0.59; P < 0.001), but was similar to those with obese NAFLD (Table 2, P = 0.17). This result suggests that systemic insulin resistance induced by MS may be an explanation for increased risk of CKD even in non-obese NAFLD. Meanwhile, the steatotic and inflamed liver itself has been known to contribute to kidney injury in NAFLD^28,29^. In NAFLD and CKD, fetuin-A, a hepatokine, promotes endothelial dysfunction and vascular wall inflammation of hepatic and glomerular endothelial cells, providing evidence for the role of an inflamed liver^28,29^. When subjects were cross-categorized based on the combination of MS and NAFLD in this study, the incidence rate of CKD was lowest in the non-MS and non-NAFLD groups (15.9%), followed by the group with NAFLD but no MS (18.7%), the group with MS but no NAFLD (27.8%), and was highest in patients with both MS and NAFLD (29.0%) (Supplementary Fig. 1). Although this result suggests the effect of MS overwhelms the effect of NAFLD, because we did not measure hepatokines, we did not determine the role of an inflamed liver on CKD development in this study. Future studies are necessary to clarify the pathogenesis and mechanism by which non-obese NAFLD contributes to a higher risk of incident CKD.

Another main finding was that decreased BW and WHR significantly reduced the risk of incident CKD even in non-obese NAFLD patients. Although numerous studies indicate that significant and sustained weight reduction is beneficial to NAFLD development and disease progression^22,23^, few studies evaluate the impact of weight loss on kidney function in the NAFLD population^20^. A *post hoc* analysis of non-alcoholic steatohepatitis patients demonstrated that those with a significant weight loss (>5%) were more likely to have improved or stabilized eGFR at the end of a 52-week life style modification training^20^. In the current study, patients with TA-% BW change ≤ −5% during follow-up showed roughly half the risk for CKD development and the benefit of weight loss was also significant in the non-obese NAFLD group. Previous studies suggest that weight loss may be helpful even in non-obese NAFLD^9,10,15,25^. One possible explanation for these findings is a reduction in visceral fat^9,10,13^. In this regard, it is noteworthy that decreased WHR, a useful anthropometric index of central obesity, was significantly associated with lower risk of incident CKD even in non-obese NAFLD patients in the present study. Obesity, especially central obesity, increases various pro-inflammatory molecules such as adipokines, interleukin-6, and tumor necrosis factor alpha, leading to the development of systemic insulin resistance^26,27^. Systemic insulin resistance is known to be an important factor in the pathogenesis of CKD^30^. From these findings, we hypothesized that insulin resistance may change according to WHR or BW changes. In this study, HOMA-IR, a surrogate of insulin resistance, decreased in the reduced WHR group, but increased in the minimal change group and the increased WHR group during the follow-up period. Therefore, we surmised that improvement in systemic insulin resistance caused by decreased abdominal fat might contribute to the reduced risk of CKD development in the reduced WHR groups, even in the non-obese NAFLD population. Moreover, considering the hazard ratio for CKD development, risk reduction due to decreased WHR outweighed those from BW loss in our study. Thus, we speculated that reducing abdominal fat strategies might be beneficial for improving kidney outcomes in these populations. Another potential mechanism may be through improvement of NAFLD *per se* induced by weight loss. Jin *et al*.^25^ reported that a 5% reduction in weight was significantly associated with steatosis improvement in non-obese NAFLD patients. Unfortunately, because liver biopsy was not available in our study, the effect of changes on liver histology cannot be determined. Future studies evaluating the association between histologic improvement and central obesity and their effects on long-term kidney outcomes are worthy of investigation.

This study has several limitations. First, we determined the presence of NAFLD using a prediction model rather than liver imaging (ultrasonography) or liver biopsy. Because a non-invasive prediction model of liver fibrosis cannot completely reflect liver inflammation or fibrosis of NAFLD on a histologic exam, this finding should be considered cautiously. Because this study was a community-based cohort study that included the general population, the high cost of liver imaging and the invasiveness of liver biopsy limited their application in our study participants. Therefore, NAFLD was determined using the NAFLD liver fat score, which is a non-invasive and validated index that has been replicated in previous studies^31^. Although lack of liver imaging or biopsy is a limitation of this study, using a prediction model may be more valuable in translating our findings to real world practice. Second, the WHR and BW changes observed in our study may not have been intentional. We could not definitively determine whether loss or gain of WHR and BW was intentional or spontaneous, due to the observational nature of this study. Therefore, therapeutic WHR or BW reduction to prevent CKD should be further tested in a prospective randomized controlled study. In the present study, we included various confounders in multivariable models and subgroup analyses were performed. When mean arterial pressure and liver fibrosis score, significant risk factors for CKD, were added to multivariable models, risk reduction from decreased WHR or BW was not changed (Supplementary Table 4). Subgroup analyses also showed consistent results, but not in diabetic patients. We suggest our small number of diabetic patients limits this study’s statistical power. However, residual confounding effects cannot be totally excluded. We cannot clarify the effect of lifestyle modification, medication, or bariatric surgery on weight changes in this study. Nevertheless, this study includes strongly convincing data, obtained from a large general population in a well-examined longitudinal study. The small number of subjects and short follow-up period of previous studies limited their ability to ascertain clinically significant kidney outcomes. Inclusion of proteinuria data and a relatively long follow-up of 12 years allowed us to ascertain the risk of adverse kidney outcomes thoroughly. Lastly, to the best of our knowledge, this is the first study to provide evidence for the beneficial effect of reduced WHR on long-term adverse kidney outcomes in non-obese NAFLD patients.

In conclusion, non-obese NAFLD patients were at a high risk of adverse kidney outcomes. A decrease in WHR, more than an average of 5% during the follow-up, was associated with significant risk reduction of CKD development in NAFLD patients, even though they were not obese. Based on these findings, we speculate that reducing abdominal fat may be a beneficial strategy to attenuate the increased risk of adverse kidney outcomes in NAFLD patients. Therefore, education for lifestyle modification and serial monitoring of WHR and kidney function should be prioritized in the management of NAFLD patients, even if they are non-obese.

## Methods

### Study design and participants

Participants from the Ansung-Ansan Cohort of the Korean Genome Epidemiology Study (KoGES) were screened in this study. The KoGES is a community-based, prospective cohort study that includes 40- to 69-year-old Korean participants from the general population who lived in the rural Ansung or urban Ansan communities from 2001 to 2002. After a baseline survey, follow-up surveys were performed every 2 years, and the latest (6^th^) follow-up was performed between 2013 and 2014. The detailed design and methods of the cohort have been previously described^32^. Among the initially enrolled 10,030 participants, a total of 6,137 participants were included in the current study (Supplementary Fig. 2). This study was conducted in accordance with the Declaration of Helsinki. The study protocol was approved by the ethics committee of the Korean Center for Disease Control and the Institutional Review Board of CHA Bundang Medical Center. All participants provided written informed consent before entering the study.

### Data collection

Demographics, clinical, and laboratory data were retrieved from the electronic database. Demographics including age, sex, socioeconomic status, medical history, and lifestyle factors such as smoking and alcohol consumption were recorded during the baseline survey and every follow-up examination using a standardized questionnaire administered by a well-trained research coordinator. A 12-h fasting blood sample and the first-voided urine were analyzed to measure biochemical variables at the central laboratory. HOMA-IR was calculated^33^. MS was defined based on the modified National Cholesterol Education Program’s Adult Treatment Panel III criteria^34^. The Chronic Kidney Disease Epidemiology Collaboration equation was used to calculate eGFR^35^.

### Assessment of changes in body weight and WHR

Anthropometric indices (height, BW, waist circumference, and hip circumference) were measured as recommended by the World Health Organization^36^. BMI was calculated as weight/height^2^ (kg/m^2^) and WHR was calculated as waist circumference/hip circumference. Changes in BW and WHR were assessed using the percent change from baseline^37^. Percentage change of BW and WHR was calculated with the following equation: [(BW or WHR at each follow-up/BW or WHR of baseline)–1] × 100. To test the effect of changes during the follow-up, TA-% BW change and TA-% WHR change were calculated for each patient.

### Follow-up and outcome

Participants were re-evaluated every 2 years with a site visit. The primary outcome was development of CKD, defined as an eGFR of <60 mL/min/1.73m^2^ and/or proteinuria of more than 1+ on the dipstick, whichever occurred first. Patients who were lost to follow up were censored at the date of the last examination (248 at the 2nd, 339 at the 3rd, 224 at the 4th, 306 at the 5th, and 436 at the 6th visit).

### Statistical analysis

Statistical analysis was performed using R (R Foundation for Statistical Computing, Vienna, Austria; www.r-project.org). Continuous variables were expressed as the mean ± standard deviation or as the median (interquartile range), and categorical variables were expressed as a number (percentage). Participants were categorized as non-obese (BMI < 25 kg/m^2^) and obese (BMI ≥ 25 kg/m^2^) groups by the recommendation for Asian and Pacific populations^38^ and the clinical practice guidelines from the Korean Society for the Study of Obesity^39^. The presence of NAFLD was defined as a NAFLD liver fat score of ≥ –0.640^31^ and FIB-4 score was calculated in the NAFLD patients^40^ (Supplementary Table 5). Baseline characteristics were compared using a Student’s *t* test or Mann-Whitney *U* test for continuous variables and a χ^2^ test for categorical variables between non-obese NAFLD and obese NAFLD or non-obese controls to assess metabolic characteristics of non-obese NAFLD patients. Cox regression analyses were performed to determine the independent risk of non-obese NAFLD for CKD development. Multivariable Cox regression models included significant variables from univariate analyses including age, sex, education, income, smoking status, diabetes mellitus, hypertension, dyslipidemia, history of cardiovascular disease, C-reactive protein concentration, and baseline eGFR. To test the prognostic impact of changes in abdominal fat and BW on CKD development in NAFLD participants, 1,563 NAFLD patients were categorized into three groups according to TA-% WHR change and TA-% BW change (≤−5%, >−5% to <5%, and ≥5%), respectively. Multivariable Cox regression analyses were performed for all NAFLD patients and subsequent stratified analyses were performed for non-obese NAFLD and obese NAFLD patients. Furthermore, subgroup analyses according to diabetes mellitus, hypertension, and HOMA-IR concentrations were also performed. To explore the association of WHR or BW changes with insulin resistance, HOMA-IR values over time were compared among the TA-% WHR change and TA-% BW change groups using a mixed-effects model. The average slope of HOMA-IR changes during the follow-up was also calculated (ΔHOMA-IR per year). A P value less than 0.05 was considered statistically significant.

## Supplementary information

Supplemental information.

## Data Availability

The datasets used for the current study are available from the corresponding author on reasonable request.
