# Supplementary material for: Decrease in waist-to-hip ratio reduced the development of chronic kidney disease in non-obese non-alcoholic fatty liver disease
Source: Sci Rep. 2020 Jun 2;10:8996. doi: 10.1038/s41598-020-65940-y (PMC7265367; doi:10.1038/s41598-020-65940-y)
Supplement: Supplementary file 1 — Supplemental information. [file 41598_2020_65940_MOESM1_ESM.pdf]

Decrease in waist-to-hip ratio reduced the development of chronic kidney disease in non-obese non-alcoholic fatty liver disease

Young Eun Chon[1], Hyung Jong Kim[2], Yu Bum Choi[2], Seong Gyu Hwang[1], Kyu Sung Rim[1], Mi Na Kim[1], Joo Ho Lee[1], Yeonjung Ha[1], and Mi Jung Lee[2*]

[1]Division of Gastroenterology, Department of Internal Medicine, Institute of Gastroenterology, CHA Bundang Medical Center, CHA University, Seongnam, Korea

[2]Department of Internal Medicine, CHA Bundang Medical Center, CHA University, Seongnam, Korea

*Corresponding author: alwyd8025@gmail.com

<u>Corresponding Author</u>:

Mi Jung Lee, M.D.

Department of Internal Medicine, CHA Bundang Medical Center,

CHA University College of Medicine, Seongnam, Republic of Korea

59 Yatap-ro, Bundang-gu, Seongnam, Gyeonggi-do, Korea, 13496

Tel: 82-31-780-1925

Fax: 82-31-780-5305

E-mail: alwyd8025@gmail.com

Supplementary Table 1. Comparison of metabolic profiles between non-obese control and non-obese NAFLD

	Non-obese control (n=3,095)	Non-obese NAFLD (n=459)	P
Age, years	51.1 ± 8.8	53.9 ± 8.5	<0.001
Men, n (%)	1,425 (46.0%)	213 (46.4%)	0.9
Diabetes mellitus, n (%)	32 (1.0%)	67 (14.6%)	<0.001
Hypertension, n (%)	202 (6.5%)	86 (18.7%)	<0.001
Metabolic syndrome, n (%)	314 (10.1%)	372 (81.0%)	<0.001
BMI, kg/m^2	22.4 ± 1.8	23.3 ± 1.4	<0.001
WHR	0.87 ± 0.03	0.89 ± 0.03	<0.001
MAP, mmHg	89.6 ± 12.1	97.4 ± 11.5	<0.001
AST, IU/L	25.0 (22.0-29.0)	29.0 (25.0-37.0)	<0.001
ALT, IU/L	19.0 (16.0-25.0)	29.0 (22.0-45.0)	<0.001
Total bilirubin, mg/dL	0.6 ± 0.3	0.6 ± 0.3	<0.001
GGT, U/L	21.0 (11.0-23.0)	25.0 (14.0-53.0)	<0.001
Fasting glucose, mg/dL	82.9 ± 13.6	93.4 ± 31.7	<0.001
HOMA-IR	1.28 ± 0.59	2.42 ± 1.97	<0.001
Total cholesterol, mg/dL	186.3 ± 33.1	194.4 ± 35.8	<0.001
HDL-C, mg/dL	47.0 ± 10.0	40.6 ± 9.7	<0.001
LDL-C, mg/dL	117.3 ± 31.4	115.8 ± 35.6	0.35
Triglyceride, mg/dL	128.9 ± 66.6	220.6 ± 154.2	<0.001
CRP, mg/dL	0.11 (0.05-0.19)	0.16 (0.08-0.27)	<0.001
eGFR, mL/min/1.73m^2	93.9 ± 12.9	93.2 ± 12.4	0.25

Note: Data are expressed as the mean ± standard deviation, median (interquartile range), or number of patients (percent).

Abbreviations: ALT, alanine aminotransferase; AST, aspartate aminotransferase; BMI, body mass index; CRP, C-reactive protein; eGFR, estimated glomerular filtration rate; GGT, gamma glutamyl transferase; HDL-C, high-density lipoprotein cholesterol; HOMA-IR, homeostatic model assessment of insulin resistance; LDL-C, low-density lipoprotein cholesterol; MAP, mean arterial pressure; NAFLD, non-alcoholic fatty liver disease; WHR, waist-to-hip ratio.

Supplementary Table 2. Categorization of NAFLD patients according to TA-% WHR change and TA-%BW change

	All NAFLD (n=1,563)	Non-obese NAFLD (n=459)	Obese NAFLD (n=1,104)
TA-%WHR change			
≤-5%	131 (84%)	28 (6.1%)	103 (9.3%)
>-5% to <5%	1,200 (76.8%)	351 (76.5%)	849 (76.9%)
≥5%	232 (14.8%)	80 (17.4%)	152 (13.8%)
TA-%BW change			
≤-5%	479 (30.6%)	122 (26.6%)	357 (32.3%)
>-5% to <5%	940 (60.1%)	279 (60.8%)	661 (59.9%)
≥5%	144 (9.2%)	58 (12.6%)	86 (7.8%)

Abbreviations: NAFLD, non-alcoholic fatty liver disease; TA-%BW change, time-averaged percent weight change; TA-%WHR change, time-averaged percent waist-to-hip ratio change.

Supplementary Table 3. Changes in HOMA-IR according to TA-% WHR change and TA-% BW change categories

	ΔHOMA-IR per year		
	All NAFLD	Non-obese NAFLD	Obese NAFLD
TA-% WHR change			
≤-5%	-0.04 (-0.09 to 0.02)	-0.02 (-0.05 to 0.02)	-0.04 (-0.11 to 0.02)
>-5% to <5%	0.04 (0.01 to 0.06)	0 (-0.05 to 0.04)	0.06 (0.03 to 0.09)
≥5%	0.19 (0.05 to 0.28)	0.15 (-0.08 to 0.39)	0.18 (0.05 to 0.31)
[1]$P_{(group \times time)}$	<0.001	<0.001	<0.001
TA-% BW change			
≤-5%	0.01 (-0.02 to 0.05)	0.03 (-0.02 to 0.07)	0.01 (-0.03 to 0.05)
>-5% to <5%	0.05 (0.01 to 0.08)	-0.03 (-0.08 to 0.03)	0.08 (0.04 to 0.12)
≥5%	0.22 (0.07 to 0.37)	0.25 (-0.07 to 0.58)	0.20 (0.07 to 0.32)
[1]$P_{(group \times time)}$	<0.001	<0.001	<0.001

Note: Data are expressed as the mean with 95% confidence interval.

[1]$P_{(group \times time)}$ was calculated by mixed-effect model.

Abbreviations: HOMA-IR, homeostatic model assessment of insulin resistance; NAFLD, non-alcoholic fatty liver disease; TA-% BW change, time-averaged percent body weight change; TA-% WHR change, time-averaged percent waist-to-hip ratio change.

Supplementary Table 4. Multivariable Cox regression analyses for risk of CKD development according to the WHR and BW changes categories in NAFLD patients

	[1]Model 1	[2]Model 2
TA-% WHR change	HR (95% CI)	HR (95% CI)
≤-5%	0.305 (0.197-0.473)	0.301 (0.194-0.466)
>-5% to <5%	1 (reference)	1 (reference)
≥5%	1.008 (0.999-1.016)	0.923 (0.702-1.215)
TA-% BW change		
≤-5%	0.579 (0.464-0.723)	0.587 (0.470-0.733)
>-5% to <5%	1 (reference)	1 (reference)
≥5%	0.837 (0.588-1.190)	0.823 (0.579-1.171)

[1]Model 1: adjusted for age, sex, education levels, income levels, smoking status, diabetes mellitus, dyslipidemia, history of CVD, CRP concentrations, baseline eGFR, and mean arterial pressure.

[2]Model 2: adjusted for age, sex, education levels, income levels, smoking status, diabetes mellitus, hypertension, dyslipidemia, history of CVD, CRP concentrations, baseline eGFR, and FIB-4.

Abbreviations: CI, confidence interval; CKD, chronic kidney disease; CRP, C-reactive protein; CVD, cardiovascular disease; eGFR, estimated glomerular filtration rate; FIB-4, fibrosis 4; HR, hazard ratio; NAFLD, non-alcoholic fatty liver disease, TA-% BW change, time-averaged percent body weight change; TA-% WHR change, time-averaged percent waist-to-hip ratio change.

Supplementary Table 5. Definition of NAFLD liver fat score and liver fibrosis score

NAFLD liver fat score	-2.89 + 1.18 × metabolic syndrome (yes = 1/no = 0) + 0.45 × diabetes (yes = 2/no = 0) + 0.15 × fasting insulin (μU/L) + 0.04 × AST (IU/L) + 0.94 × AST/ALT ratio
FIB-4 score	(Age [years] x AST [U/L]) / (Platelet count [10^9/L] x ALT [U/L]$^{1/2}$)

Abbreviations: ALT, alanine aminotransferase; AST, aspartate aminotransferase; FIB-4, fibrosis-4; NAFLD, non-alcoholic fatty liver disease.

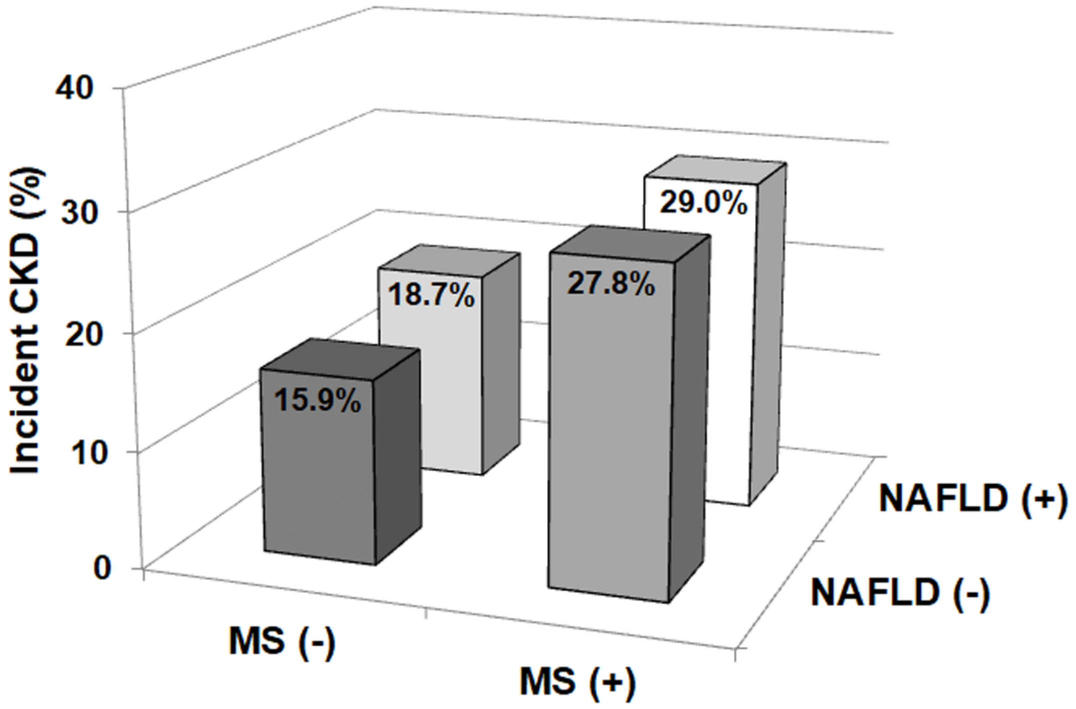

Supplementary Figure 1. Incidence rate of CKD in the 4 groups based on the combination of NAFLD and MS. Abbreviations: CKD, chronic kidney disease; MS, metabolic syndrome; NAFLD, non-alcoholic fatty liver disease.

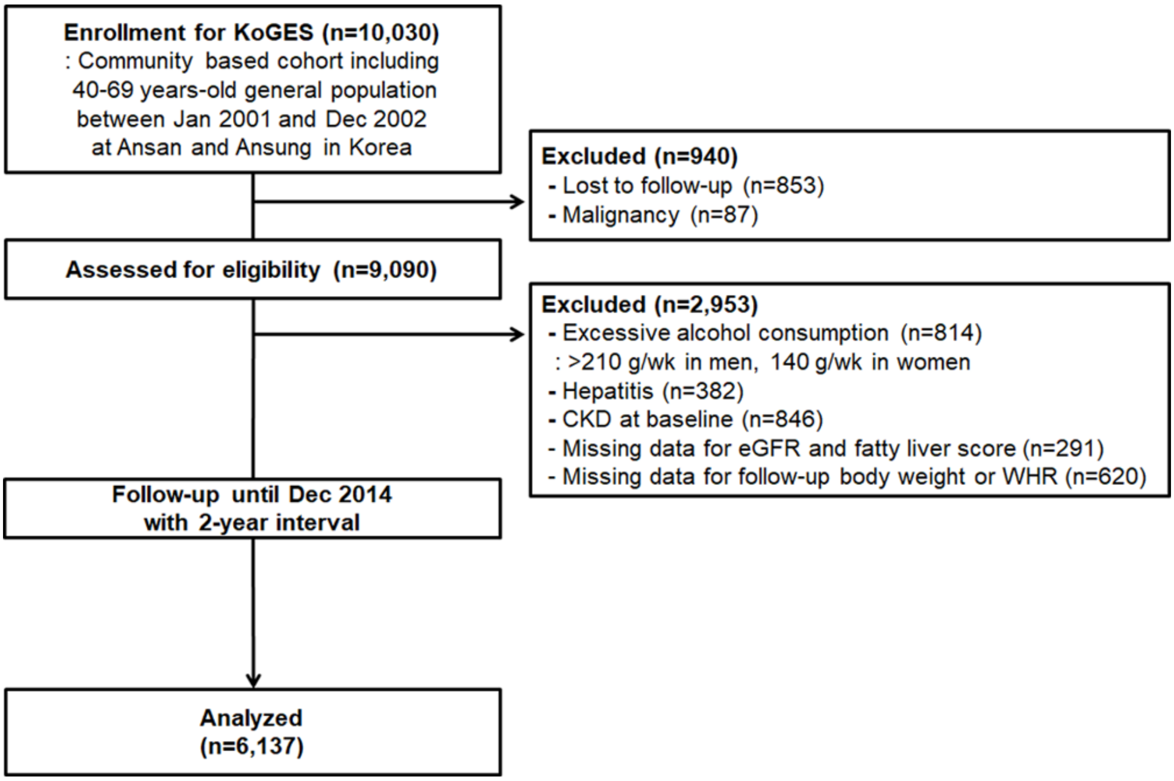

Supplementary Figure 2. Incidence rate of CKD in the 4 groups based on the combination of NAFLD and MS. Abbreviations: CKD, chronic kidney disease; MS, metabolic syndrome; NAFLD, non-alcoholic fatty liver disease.
